# Cement-Induced Coagulation of Aqueous Graphene Oxide with Ultrahigh Capacity and High Rate Behavior

**DOI:** 10.3390/nano8080574

**Published:** 2018-07-27

**Authors:** Xiaoya Yuan, Jiawei Niu, Junjie Zeng, Qiuye Jing

**Affiliations:** College of Materials Science and Engineering, Chongqing Jiaotong University, Chongqing 400074, China; Niujw2018@163.com (J.N.); 18773156035@163.com (J.Z.); m15922871980@163.com (Q.J.)

**Keywords:** graphene oxide, cement, coagulation, ultrahigh capacity

## Abstract

Graphene oxide (GO) has excellent physicochemical properties and is used in multiple areas. However, the potential toxicity and environmental problems associated with GO increase its risk to the ecological system. In this study, cement was employed as a coagulant to eliminate GO from aqueous solutions. The effects of the cement dosage, the contact time, and the concentration and volume of the aqueous GO solution on the GO coagulation capacity were investigated in detail. The results showed that the dosage of cement had a significant effect on the coagulation process, and coagulation equilibrium was achieved in less than 1 h. Compared to coagulants used to remove GO from water in other reports, cement exhibited an ultrahigh coagulation capacity of approximately 5981.2 mg/g with 0.4 mg/mL GO solution. The kinetic analysis showed that the GO removal behavior could be described by a pseudo second-order model. The in-depth mechanism of GO coagulation using cement included Ca^2+^-induced coagulation of GO and adsorption by the hydrated product of cement paste. The present study revealed that cement could be a very cheap and promising material for the efficient elimination of GO from aqueous solutions.

## 1. Introduction

Graphene oxide (GO) is one of the most important graphene derivatives and has many unique features, such as excellent mechanical properties, high surface functionalization, high surface area and good dispersibility in aqueous solutions [1]. GO is used in multiple areas, such as water treatment [2], nanosensors [3], supercapacitors [4] and nanomedicine [5,6,7]. It has abundant epoxy and hydroxyl functional groups at its basal plane, as well as carbonyl and carboxyl groups situated at the edges [8]. Due to the large number of hydrophilic groups within its structure, GO has good wettability and surface activity, and can be dispersed in water to form a stable colloidal suspension; however, parts of GO are inevitably released into the natural environment during its manufacture and use. There are many concerns about its ecological security as GO has recently been demonstrated to be one of the most toxic graphene-based materials [9]. Souza et al. evaluated GO effects with an effective concentration during acute exposure of 1.25 mg/L of GO. Chronic exposure resulted in a significant decrease in the number of neonates [10] and GO was also highly toxic to one of the most common fresh water ciliate protists, *paramecium caudatum*, at low concentrations [11]. The potential toxicity and high mobility of GO can cause serious long-term cumulative toxic effects in living organisms, and it has been demonstrated that GO and its derivatives can accumulate in the lung, liver and spleen for a long time [12,13,14]. Unlike organic pollutants, GO is not easily converted into harmless end products, or degraded to non-toxic small molecules, and tends to aggregate in living organisms with strong van der Waals forces [15,16]. Therefore, it is urgent that GO be removed from the natural aquatic environment.

During the past decade, many technologies have been applied to deal with various pollutants (e.g., organic dyes and heavy metal ions), including ion exchange [17,18], adsorption [19,20], coagulation [21], chemical precipitation [22] and membrane separation [23]. In comparison with these technologies, coagulation has been considered a promising potential method for the removal and aggregation of GO or other nanomaterials. For instance, Wang et al. reported that Mg/Al/La-CLDHs and Ca/Al/La-CLDHs exhibited maximum GO removal capacities of 565.8 and 558.6 mg/g, respectively, with the coagulation of GO on La-doped LDH mainly dominated by electrostatic attraction and hydrogen bonding [24,25,26,27,28]. A study by Yuan et al. found that calcined MgAl-layered double hydroxides (MgAl-LDH) showed moderate removal capacity of 984.2 mg/g towards GO, due to the memory effect of calcined LDH [20]. However, these methods for removing GO are slow and time-consuming because of the dominance of physical absorption or coagulation in most cases. Additionally, the coagulants usually used to remove GO are expensive and have low removal capacity. Therefore, it is necessary to develop low-cost and highly efficient coagulants to remove GO from the aquatic environment.

Cement is the key component of concrete and mortar, which are two of the most important and widely used building materials. Cement gradually congeals and hardens upon contact with water. In this process, cement clinker minerals undergo a series of complex chemical reactions with water to generate hydrous calcium silicate gel (C-S-H) and calcium lanthanum, Ettringite crystal (AFt), calcium hydroxide crystals (CH), and monoculture calcium aluminosilicate (AFm) crystals [27]. Furthermore, C-S-H gels have extremely high specific surface energy and ion exchange capacity, and can be chemically displaced through adsorption, symbiosis, and intercalation. Using methods such as curing foreign ions, Ettringite can also be replaced by chemical substitution in the crystal column, and the channel contains many external ions [28]. Moreover, a large amount of OH^−^ is released during the cement hydration process, which makes the cement paste highly basic. In this study, cement was employed to remove GO from an aqueous solution, and the effects of the cement dosage, the GO concentration and volume, and the amount of hydrated cement paste prepared with different contact times, on GO coagulation behavior were investigated in detail.

## 2. Experimental Section

### 2.1. Materials

Cement was purchased from Huaying TCC Cement Co., Ltd. (Chongqing, China). The cement type was ordinary silicate P.O. 42.5R, which was used directly without further purification. The GO was provided by the Chongqing Institute of Green and Intelligent Technology, Chinese Academy of Sciences. Prior to experiments, the GO was dispersed into ultrapure water to prepare a stock suspension (0.2 mg/mL, 0.4 mg/mL and 0.6 mg/mL) at room temperature and was used in the following experiments.

### 2.2. Characterization

The samples were characterized by X-ray diffraction (XRD) using a Brooke D8 diffractometer at a voltage of 40 kV and a current of 40 mA, with Cu Kα radiation (*k* = 1.541 Å), to analyze the crystal structure. The XRD pattern was recorded with a scanning rate of 0.1°s^−1^ in the 2θ ranging from 5° to 100°. Scanning electron microscopy (SEM) images were obtained with a ZEISS SIGMA 300 field emission scanning electron microscope, with acceleration voltage of 30 kV. High-resolution transmission electron microscopy (HRTEM) was performed on a FEI Tecnai G2 F20 field-emission transmission electron microscopy, at an accelerating voltage of 200 kV. X-ray photoelectron spectroscopy (XPS) measurements were performed on a Thermo Fisher ESCALAB 250XI photoelectron spectrometer using monochromatic Al Kα X-ray source (hv = 1486.6 eV). Raman scattering measurements were carried out at room temperature with a LabRAM HR Evolution micro-Raman spectrometer (HORIBA Jobin Yvon S.A.S, Paris, France).

### 2.3. Coagulation Tests

The batch coagulation experiments using cement were carried out in conical flasks of 500 mL or 1000 mL, at a constant magnetic stirring speed of 1000 rpm. The typical procedure was as follows: a certain amount of cement was added to the pre-prepared GO solution and, at given time intervals, 5 mL of the aliquot was withdrawn, centrifuged, and the supernatant GO concentration was monitored by UV-Vis spectrometer at the wavelength of 227 nm. The effects of the dosage of cement and GO concentration, on the removal efficiency of GO were investigated.

According to the blank tests, the coagulation of GO onto the transparent bottle-wall was negligible. So the amount of GO coagulated by cement was calculated from the difference between the initial concentration (*C*_0_) and the equilibrium concentration (*C_e_*). The coagulation capacity (*q_e_*) of GO onto cement and the removal percentage (*R*), were calculated using the following equations:

(1) $$q_{e}=\frac{\left(C_{0}-C_{e}\right)V}{m}$$

(2) $$R=\frac{C_{0}-C_{e}}{C_{0}}\times 100\%$$

where *q_e_* (mg/g) was the amount of GO adsorbed at equilibrium, *C*_0_ and *C_e_* (mg/mL) were the initial and equilibrium concentrations of GO in the solution, *V* was the volume of the suspension (mL), *m* was the mass of cement (g), and *R* meant the removal percentage of GO.

## 3. Results and Discussion

### 3.1. Batch Coagulation Experiments

**Figure 1:**
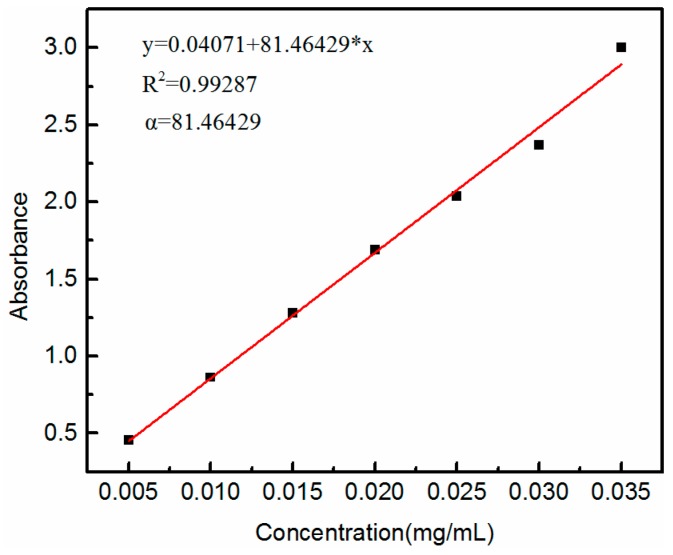
The correlation between graphene oxide (GO) concentrations and its UV absorbance intensity at 227 nm.

**Figure 2:**
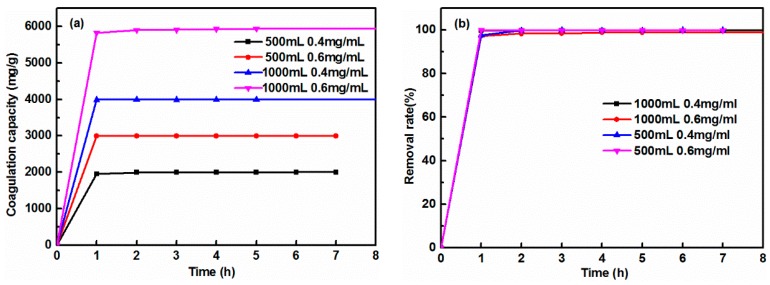
(**a**) Coagulation capacity, and (**b**) removal rate of GO vs. contact time using 0.1 g cement.

**Figure 3:**
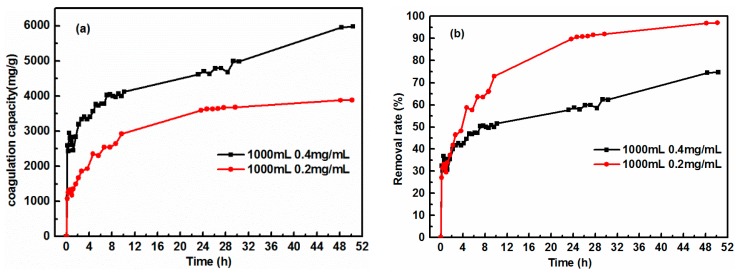
(**a**) Coagulation capacity, and (**b**) removal rate of GO vs. contact time using 0.05 g cement with the GO volume of 1000 mL, 0.2 and 0.4 mg/mL, respectively.

Cement dosage is an important factor affecting the coagulation property of GO, due to the active sites and effective contact areas of cement, and should be considered in low-cost practical environmental remediation [29]. Before conducting the GO coagulation experiments, one must consider how to precisely monitor the concentration of GO solution during the whole coagulation process. Several control experiments were tried and it was found that the adsorption intensity of aqueous GO solution at 227 nm obeys Beer-Lambert Law at GO concentration below 0.03 mg/mL (Figure 1) [21]. The absorption coefficient (α) for GO at the wavelength of 227 nm was 81.46429 L/(g·cm). In order to obtain the removal rate of cement for GO, a well-designed experiment to achieve equilibrium of coagulation using low sorbent loading and high GO concentration (0.4 and 0.6 mg/mL) was attempted. Figure 2 shows the variation of coagulation capacity and removal percentage of GO, using different volumes (500 and 1000 mL) and concentrations (0.4 and 0.6 mg/mL) of GO solution, and 0.1 g cement. Unexpectedly, cement could completely remove GO within one hour, indicating that cement exhibited superfast coagulation behavior toward aqueous GO. In order to obtain the equilibrium coagulation capacity of cement for GO, further reductions in the dosage of cement were conducted. Figure 3 presents the effect of contact time on the coagulation capacity of cement with 0.2 and 0.4 mg/mL GO concentrations and 0.05 g cement. As the contact time was extended, the coagulation capacity gradually increased. Note that the coagulation rate was very fast within the first period of 8 h, then gradually slowed down and plateaued after 32 h, suggesting that coagulation equilibrium was achieved within 50 h. Meanwhile, when the dosage of cement was 0.05 g, a significantly high GO coagulation capacity of 3884.4 and 5981.2 mg/g was successfully achieved with GO concentrations of 0.2 and 0.4 mg/mL, respectively.

According to the recent literature surveyed, several adsorbents/coagulants have been used to remove GO, and these are listed in Table 1. Compared to the findings reported in other papers, it is clear that the cement in our study exhibited superhigh coagulation capacity with a very fast removal rate, as discussed above. Moreover, cement, as a common building material, is cheaper than other coagulants/adsorbents, whose price is about $57 per ton. In brief, cement may be the most promising coagulant for the removal of GO from water.

### 3.2. Coagulation Kinetics

**Figure 4:**
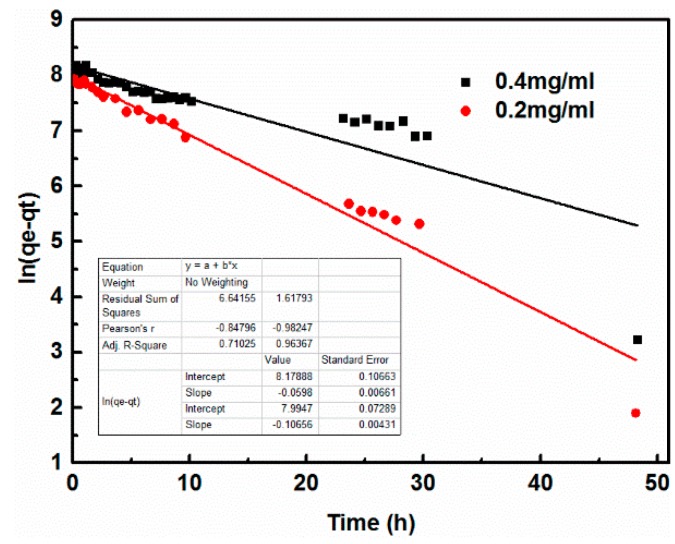
Pseudo first-order kinetics with different GO concentrations.

**Figure 5:**
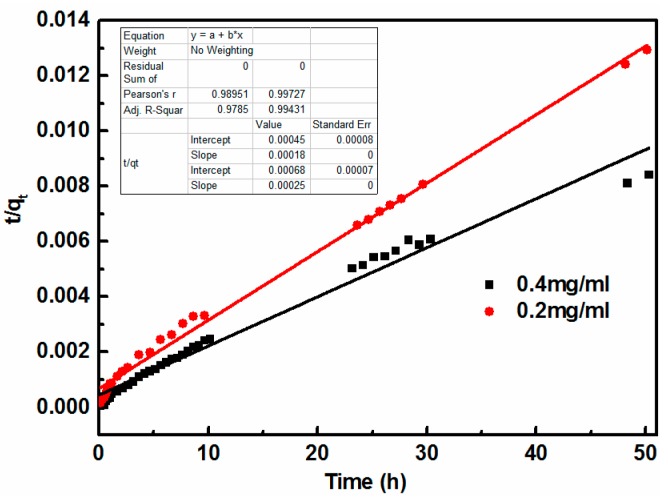
Pseudo second-order kinetics with different GO concentrations.

Coagulation kinetics were investigated to better understand the mechanism of GO coagulation. The experimental data were modeled using the pseudo first-order and pseudo second-order models to evaluate the coagulation kinetics of GO. Pseudo-first-order (Equation (3)) and pseudo-second-order (Equation (4)) models [20] were used to fit the coagulation data as follows:

(3) $$\ln\left(q_{e}-q_{t}\right)=\ln q_{e}-k_{1}t$$

(4) $$\frac{t}{q_{t}}=\frac{1}{k_{2}q_{e}^{2}}+\frac{t}{q_{e}}$$

where *q_e_* and *q_t_* (mg/g) are the coagulation capacity of GO at equilibrium and at times *t*, respectively. The values of *q_e_* and *k*_1_ can be determined from the intercept and slope of the linear plot of ln(*q_e_* − *q_t_*) vs. *t* where *k*_1_ (1/h) is the rate constant of the pseudo first-order and *k*_2_ (g/mg·h) is the rate constant of the pseudo second-order coagulation. The values of *q_e_* and *k*_2_ can be obtained from the slope and intercept of the plots of *t*/*q_t_* against *t* as shown in Figure 4 and Figure 5.

Table 2 lists the kinetic parameters calculated from the linear regression for the pseudo first-order equation (*q_e_*, *k*_1_) and the pseudo second-order kinetic model (*q_e_*, *k*_2_). For each case, the corresponding correlation coefficients *R*^2^ are also shown.

The comparison between the experimental coagulation capacity (*q_exp_*) value and the calculated coagulation capacity (*q_cal_*) value showed that the *q_cal_* value was very close to the *q_exp_* value for the pseudo second-order kinetics (Figure 5). Therefore, the GO coagulation process by cement could be approximated by a pseudo second-order model, which was further confirmed by the correlation coefficient value *R*^2^ for the pseudo second-order model (Figure 5), which was higher than that for the pseudo first-order model (Figure 4). Such a finding is also in good agreement with previous studies about wastewater treatments [31,32].

### 3.3. Structure Analysis of Cement-Induced GO Coagulation Products

In order to further understand the state of functional groups and to explore the possible coagulation mechanism, a number of characterizations and control experiments were performed.

**Figure 6:**
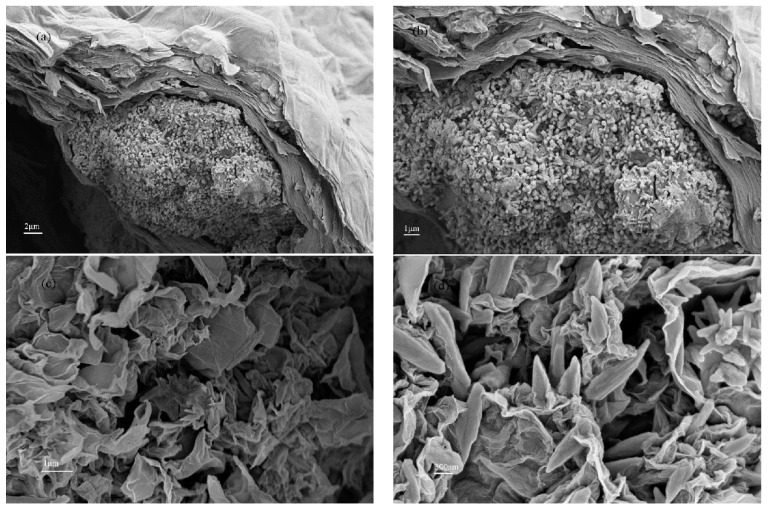
SEM images recorded for coagulation products of GO by cement (CPGC) for 10 h with different scale bar (**a**) 2 μm; (**b**) 1 μm; (**c**) 1 μm; (**d**) 300 nm.

**Figure 7:**
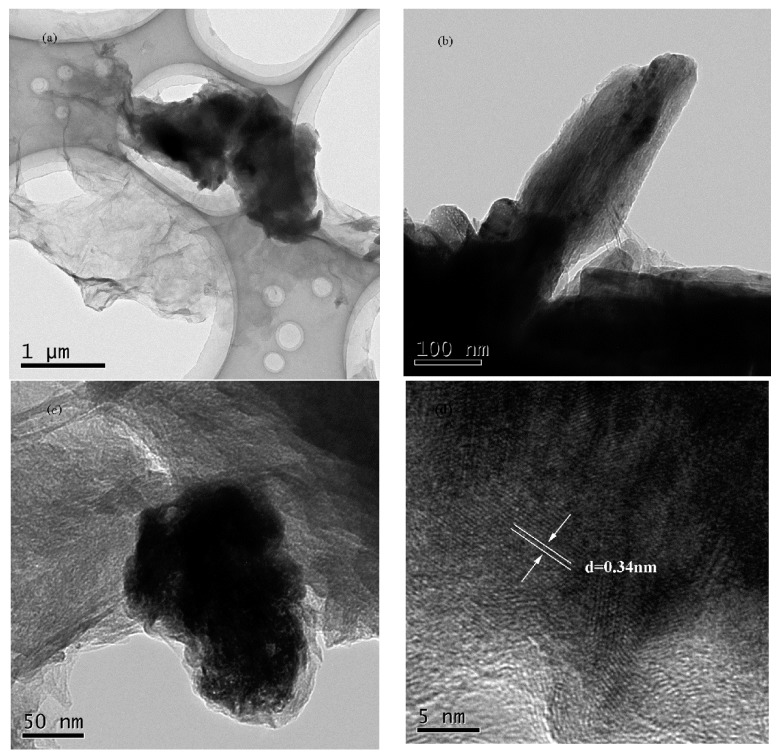
TEM images of the CPGC for 10 h with different scale bar (**a**) 1 μm; (**b**) 100 nm; (**c**) 50 nm; (**d**) 300 nm.

The morphology of the coagulation products of GO by cement (CPGC) are shown in Figure 6. The CPGC were obtained with 0.4 mg/mL GO aggregated by 0.1 g cement for 10 h. From Figure 6a,b, it can be seen that the GO were restacked together, further confirming the successful GO aggregate induced by cement. In Figure 6c,d, silk-like films, characteristic of multilayers of GO sheets, were clearly detected. Needle rod crystals were also observed, assigned to the AFt (an early hydration product of cement). Moreover, the TEM images revealing the fine-structure of the CPGC are more clearly are shown in Figure 7. The relatively transparent areas indicated the fine structure of few-layered GO nanosheets (Figure 7a), and the darker areas were due to the hydration products of cement (Figure 7b). The hydrated products of cement were clearly found to be randomly distributed on the surface of the GO nanosheets because of the large specific surface of GO [33]. The overall morphology of the CPGC composite was the relatively heterogeneous amorphous characteristic and the silk-like flakey structure (Figure 7a–c). The spacing between graphene oxide nanoplatelets was 0.34 nm (Figure 7d).

**Figure 8:**
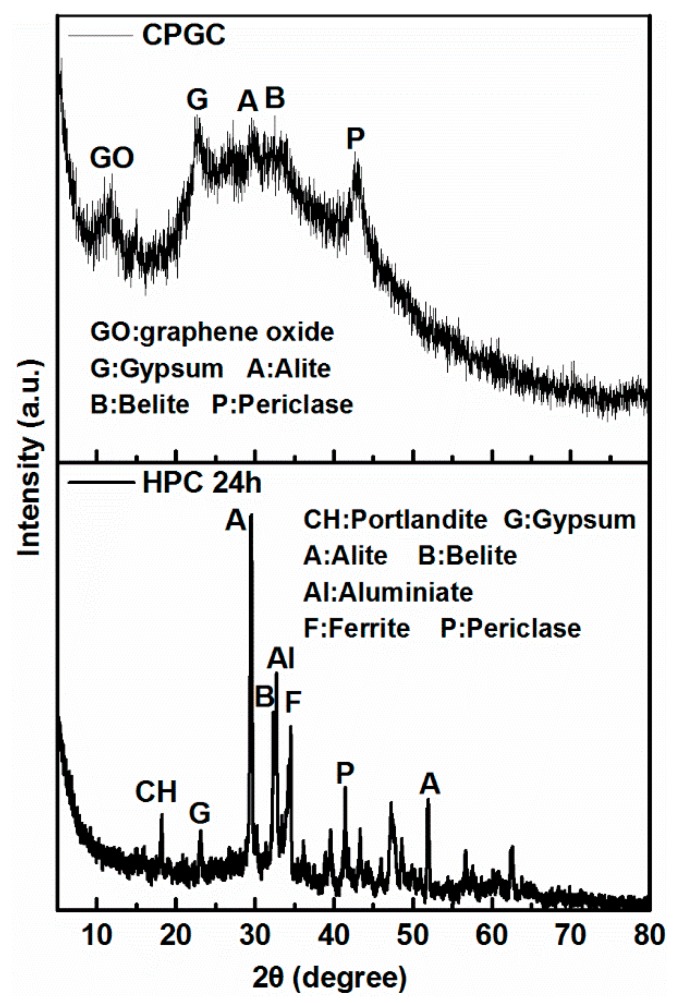
XRD patterns for CPGC and hydration products of cement (HPC).

Figure 8 depicts the XRD patterns for the GO coagulation products using cement at different contact times. For the purpose of comparison, all XRD peaks for hydration products of cement aged for 24 h (HPC 24 h, in which the same dosage of cement to distilled water was used, hydration products of cement were collected for 24 h, and hydration was terminated with anhydrous ethanol) were also marked in the XRD patterns. An initial comparison of the peak intensities between CPGC and the cement hydration products provided some insights into the composition of GO coagulation products induced by cement. During the cement hydration process, a series of complex chemical reactions between cement clinker minerals and water take place, generating C-S-H, AFt, CH and AFm [34]. These hydrated products were also found in the XRD diffraction of CPGC. In the XRD pattern, intensive peak at 2θ = 11.8° was assigned to the characteristic peak of GO, and the basal space value of (d001) was 0.34 nm, which was highly consistent with the interlayer value calculated by TEM (shown in Figure 7d), indicating that the oxygen-containing functional groups were introduced into the GO sheets [35]. Furthermore, the diffraction peaks at 2θ = 18°, 22°, 29.6°, 32°, 33°, 34°, 41.8°, 52° correspond to CH, gypsum, alite, belite, aluminate, ferrite, periclase, and alite, indicating that GO-coagulation did not affect the intrinsic hydration of the cement [36,37].

**Figure 9:**
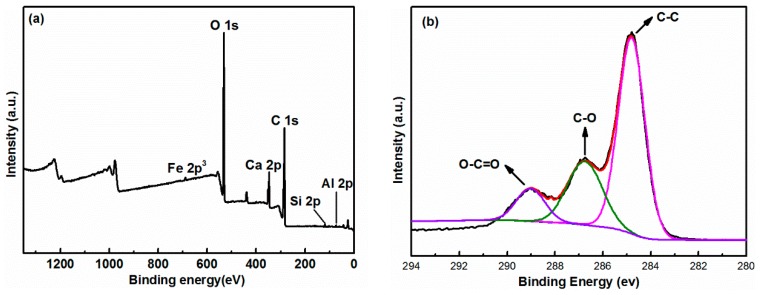
(**a**) X-ray photoelectron spectroscopy (XPS) survey spectra, and (**b**) deconvoluted C1s spectra of CGPC.

In order to give more detailed information about the coagulation mechanism, the CPGC were analyzed using XPS (Figure 9). The XPS survey spectra of the CPGC revealed that Fe, O, Ca, C, Si and Al were the predominant elements of the as-prepared product from the binding energy values of Fe 2p^3^, O 1s, Ca 2p, C 1s, Si 2p and Al 2p upon hydration of cement in aqueous GO solution, confirming the hydration products of cement and GO phases in the CPGC composite. The high-resolution C1s spectra of CPGC composite could be further deconvoluted into three components: the non-oxygenated ring C (284.8 eV); the carbon in C-O (286.7 eV); and the carboxylate carbon (O-C=O, 289.0 eV) [38], which further suggested the successful GO coagulation induced by cement.

**Figure 10:**
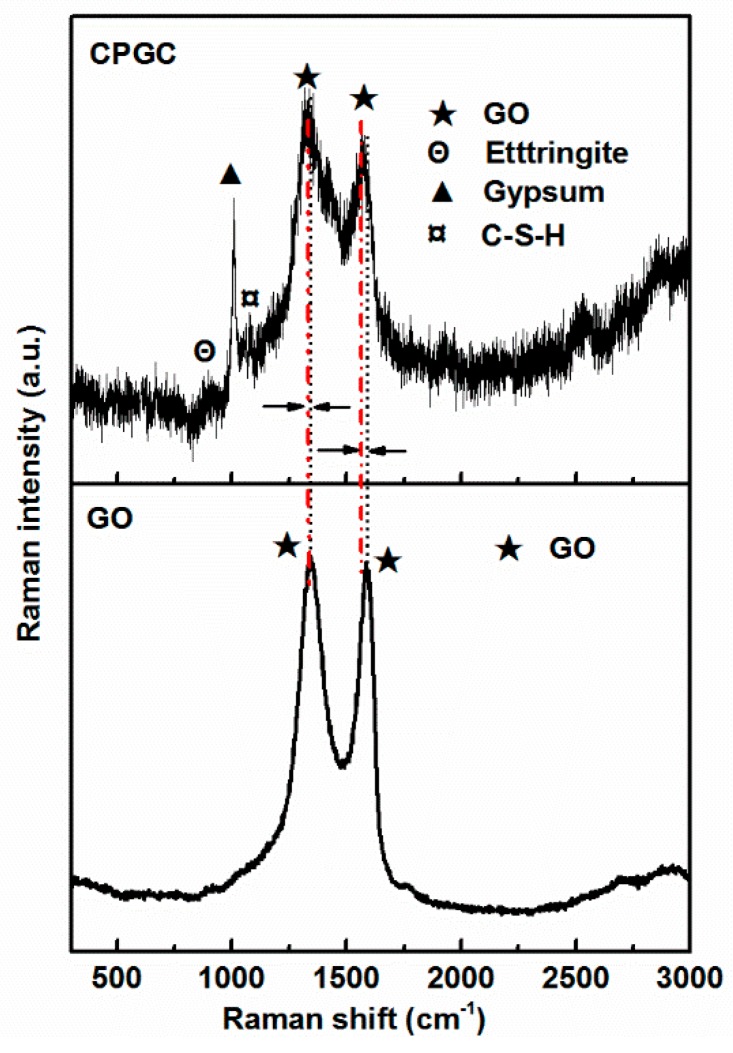
Raman spectrum of the CPGC and GO.

Figure 10 depicts the Raman spectrum of the CPGC. Raman scattering is strongly sensitive to the electronic structure, and has proved to be an essential tool for characterizing graphite and graphene materials [39,40]. Raman spectroscopy of graphene is generally characterized by two main features: the G-peak, which arises from first-order scattering of the E2g phonon from sp^2^ carbon atoms (generally observed at 1575 cm^−1^); and the D-peak (1355 cm^−1^), which arises from the breathing mode of κ-point photons of A1g symmetry [41]. Our results showed that the G-band and D-band of GO appeared at 1568 cm^−1^ and 1342 cm^−1^, respectively. Compared to those for pure GO, the G-band and D-band peaks exhibited significant blue-shift, suggesting a strong interfacial interaction between the cement hydration products and GO. The peaks of CGPC at 931, 1006 and 1137 cm^−1^ were attributed to AFt, gypsum, and C-S-H [42].

### 3.4. Coagulation Mechanism

The cement hydration process produces a large number of calcium ions, and it has been reported that Ca^2+^ has an aggressive effect on the colloidal stability of GO solution [43]. In order to clarify the role of Ca^2+^ in cement-induced coagulation of GO, a well-designed experiment was attempted to measure the Ca^2+^ concentration released by cement hydration while depositing GO with the same concentration of Ca^2+^. One gram of cement was put into 500 mL of distilled water, and the maximum amount of Ca^2+^ was 6.87 × 10^−4^ mol as measured by the ethylenediaminetetraacetic acid (EDTA) method [44]. Then, using CaCl_2_ as the Ca^2+^ source, the equal molar amount of CaCl_2_ (0.0508 g) was added to a 500 mL solution of 0.4 mg/mL GO. After being stirred for 50 h at 1000 rpm, the supernatant GO solution was collected. By measuring the concentration difference before and after the addition of CaCl_2_, Ca^2+^-induced coagulation capacity was calculated to be 3940.7 mg/g, which accounted for more than 50% in the maximum GO coagulation capacity of cement. This confirmed that Ca^2+^ decreased the stability of GO and displayed an aggressive ability in GO aggregation [45] because of the binding capacity of Ca^2+^ ions with hydroxyl and carboxyl functional groups of GO [46]. Therefore, we believe that one of the mechanisms of GO removal by cement was Ca^2+^-induced coagulation.

**Figure 11:**
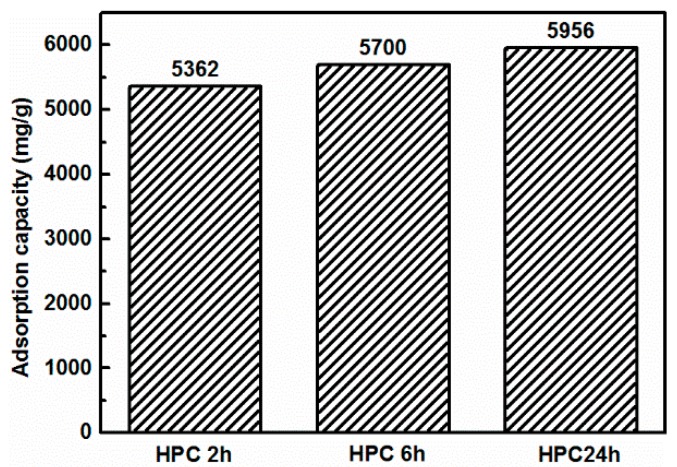
The adsorption capacity of 0.05 g HPC aged for different times (2 h, 6 h, and 24 h).

An experiment was devised in order to further elaborate the possible mechanism of GO removal caused by the physical adsorption of HPC. The cement was put into water to start a hydration reaction, and the solid samples of HPC were taken at 2, 6 and 24 h, respectively. The initial coagulation rate of GO by cement was very fast. Likewise, the amount of Ca^2+^ released was highest during the first two hours of the cement hydration process. Hence, we chose to stop the hydration at two hours and make it an adsorbent. The hydration process of each sample was terminated with anhydrous ethanol [47]. Then, adsorption experiments were conducted using 0.05 g of solid sample of the above HPC prepared at different times and 1000 mL of 0.4 mg/mL GO solution. As shown in Figure 11, it was found that the adsorption capacities were 5362, 5700 and 5956 mg/g for the HPC samples at 2, 6, and 24 h (abbreviated as HPC 2 h, HPC 6 h, HPC 24 h), respectively. The adsorption capacity of HPC gradually increased as the hydration time increased and its value was high enough that it was, to some extent, responsible for the ultrahigh capacity of GO coagulation caused by cement. Therefore, one can easily conclude that the adsorption of HPC for GO was also an indispensable mechanism involved in the cement-induced GO coagulation process described.

**Figure 12:**
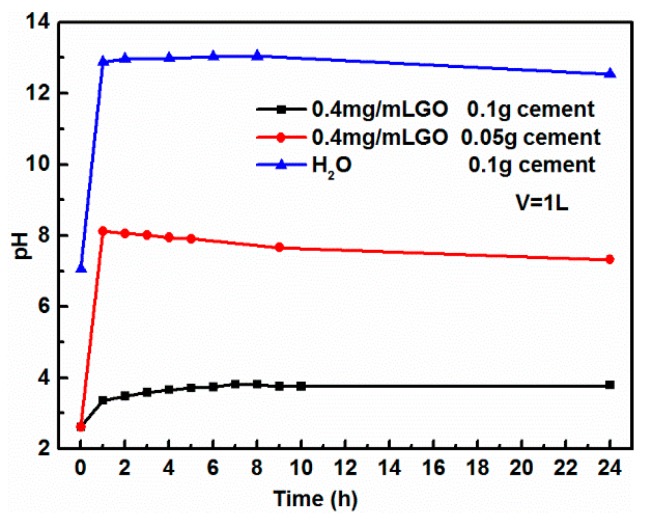
The variation of the batch solution pH by contact time during the cement-induced GO coagulation with a volume of 1000 mL.

It has been reported that the critical concentration of GO, above which the stacking interactions play a role, is 0.017 mg/mL for a phosphate buffer of pH 7.4 [7]. It can be seen in Figure 12 that the initial pH of GO was 2.62, much lower than the pH of the phosphate buffer, while GO is very stable under acidic conditions. The coagulation of GO was pH-dependent. As soon as the cement hydration began, the pH of the slurry quickly increased slightly to about 13.0 because a lot of OH^−^ were rapidly released from the surface of the cement particles [48]. As shown in Figure 12, when cement was added to the GO solution, the pH value sharply increased within 1 h. However, the pH of the mixture solution decreased slowly until stable after contact time of 24 h. Combined with the Raman analysis above, it could be inferred that there was a chemical reaction between OH^−^ produced by cement hydration and the carboxyl and epoxy groups on the surface of the GO nanosheets, which could account for the slight decrease in the solution pH at the last stage of this coagulation.

## 4. Conclusions

Cement-induced coagulation of aqueous GO exhibited ultrahigh capacity and high rate behavior. The GO removal capacity of cement was dependent on contact time, the dosage of cement, and the concentration and volume of the aqueous GO solution. This coagulation process was well-fitted by the pseudo-second-order model. The coagulation mechanism of GO on the cement included Ca^2+^-induced coagulation of GO and adsorption by the cement hydration products. The study demonstrated that cement is a promising adsorbent for the coagulation of GO from water. It is worthy of note that the GO coagulation product using cement can be further used as high-performance building material [49].

## Figures and Tables

**Table 1:** Comparisons of the coagulation capacity of graphene oxide (GO) with different coagulants.

Coagulants	Removal Capacity (mg/g)	References
Al_2_O_3_	0.59	[30]
LDH-Cl	57	[25]
Mg/Al LDH	79.9	[21]
Ca/Al LDH	123	[21]
LDO-G1	448.3	[16]
Ca/Al/La-MMO	558.8	[26]
Mg/Al/La-MMO	565.8	[26]
MgAl-MMO	984.2	[20]
Cement	5981.2	This study

**Table 2:** Kinetic parameters for GO coagulation onto cement.

Dosage (mg/mL)	*q*_e,exp_ (mg/g)	Pseudo First-Order			Pseudo Second-Order		
		*k*_1_ (min^−1^)	*q*_e,cal_ (mg/g)	*R* ^2^	*k*_2_ (g/mg·g)	*q*_e,cal_ (mg/g)	*R* ^2^
0.2	3884.4	0.10656	2965.2	0.96367	0.0000904	4035.903397	0.9943
0.4	5981.2	0.00589	3564.8	0.71025	0.0000701	5631.011104	0.9785
